# Micro-epidemiological structuring of *Plasmodium falciparum* parasite populations in regions with varying transmission intensities in Africa

**DOI:** 10.12688/wellcomeopenres.10784.2

**Published:** 2017-09-08

**Authors:** Irene Omedo, Polycarp Mogeni, Teun Bousema, Kirk Rockett, Alfred Amambua-Ngwa, Isabella Oyier, Jennifer C. Stevenson, Amrish Y. Baidjoe, Etienne P. de Villiers, Greg Fegan, Amanda Ross, Christina Hubbart, Anne Jeffreys, Thomas N. Williams, Dominic Kwiatkowski, Philip Bejon

**Affiliations:** 1 KEMRI-Wellcome Trust Research Programme, Centre for Geographic Medicine Research-Coast, Kilifi, Kenya; 2 Department of Medical Microbiology, Radboud University Nijmegen Medical Centre, Radboud Institute for Molecular Life Sciences, Nijmegen, Netherlands; 3 London School of Hygiene and Tropical Medicine, London, UK; 4 Wellcome Trust Centre for Human Genetics, University of Oxford, Oxford, UK; 5 Medical Research Council Unit, Fajara, The Gambia; 6 Johns Hopkins Bloomberg School of Public Health, Baltimore, Maryland, USA; 7 Centre for Tropical Medicine and Global Health, Nuffield Department of Medicine Research Building, University of Oxford, Oxford, UK; 8 Department of Public Health, Pwani University, Kilifi, Kenya; 9 Department of Epidemiology and Public Health, Swiss Tropical and Public Health Institute, Basel, Switzerland; 10 Department of Medicine, South Kensington Campus, Imperial College London, London, UK; 11 Wellcome Trust Sanger Institute, Wellcome Genome Campus, Hinxton, Cambridge, UK; 12 Centre for Clinical Vaccinology and Tropical Medicine, University of Oxford, Oxford, UK

**Keywords:** Plasmodium falciparum, malaria, parasite mixing, population structure, micro-epidemiological, targeted control, principal component analysis, genotyping

## Abstract

**Background: **The first models of malaria transmission assumed a completely mixed and homogeneous population of parasites.  Recent models include spatial heterogeneity and variably mixed populations. However, there are few empiric estimates of parasite mixing with which to parametize such models.

**Methods**: Here we genotype 276 single nucleotide polymorphisms (SNPs) in 5199 *P. falciparum* isolates from two Kenyan sites (Kilifi county and Rachuonyo South district) and one Gambian site (Kombo coastal districts) to determine the spatio-temporal extent of parasite mixing, and use Principal Component Analysis (PCA) and linear regression to examine the relationship between genetic relatedness and distance in space and time for parasite pairs.

**Results: **Using 107, 177 and 82 SNPs that were successfully genotyped in 133, 1602, and 1034 parasite isolates from The Gambia, Kilifi and Rachuonyo South district, respectively, we show that there are no discrete geographically restricted parasite sub-populations, but instead we see a diffuse spatio-temporal structure to parasite genotypes.  Genetic relatedness of sample pairs is predicted by relatedness in space and time.

**Conclusions**: Our findings suggest that targeted malaria control will benefit the surrounding community, but unfortunately also that emerging drug resistance will spread rapidly through the population.

## Introduction

The earliest models of malaria transmission assumed a completely mixed and homogenous parasite population ^1, 2^. However, malaria transmission is highly heterogeneous, and follows the Pareto principle where 80% of infections occur in only about 20% of the population ^3^. Consequently, there is increasing interest in models allowing for spatial heterogeneity and variably mixed populations of parasites ^4– 7^. There are now several epidemiological studies describing spatial heterogeneity of malaria on varying geographical scales ^8– 19^. This heterogeneity is characterized by infection hotspots which usually persist even after transmission has been reduced in surrounding areas ^9, 11, 20– 25^, and thus act as reservoirs of infection ^21, 26^. Achieving any meaningful reduction in transmission in regions containing malaria hotspots will require a scale up of control activities, including repeated mass administration of Artemisinin Combination Therapy (ACT) drugs, increased coverage of long lasting insecticide treated nets (LLINs) and intensive indoor residual spraying (IRS). These measures are very costly and may not be realistic for universal coverage in most of the resource-poor endemic countries. Thus, targeted control may be more important, and is likely to be required to eliminate malaria ^3, 21, 27, 28^.

Mathematical models show that targeting hotspots may reduce transmission in surrounding areas ^11, 22^. These models, however, assume that hotspots are stable and that mosquito mixing in the community is homogeneous ^22^. Studies have shown that certain species of mosquitoes exhibit some level of site fidelity, where they return to the same homesteads to feed ^29^. If such behaviour is the norm with very little mixing, then this would greatly reduce the community-wide impact of targeted interventions, and interventions would be beneficial only to individuals within the targeted region. If, however, transmission networks operate freely over large geographical areas, then these interventions would likely have an impact beyond the targeted region. Furthermore, parasite evolution takes place in a micro-epidemiological context and the spread of drug resistance or new antigenic variants through the population will also be critically dependent on the degree of mixing of parasite populations.

Few studies currently provide empiric evidence on the mixing of parasites over space and time, yet this evidence is important as parasite mixing is likely to affect the outcome of targeted control interventions ^23^. The community-wide impact of targeted control has not been studied extensively, although early controlled trials showed that bed nets were effective at reducing child morbidity and mortality associated with malaria, in villages or communities randomised to the intervention in The Gambia ^30^ and Kilifi ^31^. More recent studies have shown that the use of bed nets in a village randomized to intervention in Asembo, western Kenya, also protected individuals just outside the intervention village who were themselves not using bed nets ^32^. A cluster-randomized controlled trial on the impact of targeting integrated control measures to hotspots showed temporally limited effect on reducing transmission in areas surrounding the targeted hotspots ^23^. In order to inform future targeted control strategies more precise empiric data on parasite mixing is required.

We hypothesized that by genotyping parasites with fine-scale temporal and spatial data we would be able to determine fine-scale structure to the population and infer the degree of parasite mixing over small geographical areas which are likely to be the focus of targeted malaria control programs ^23, 27^. We used SNP genotyping of *Plasmodium falciparum* field isolates from three African sites and analysed the genetic relatedness among parasites within individual sites, in order to determine the level of parasite mixing on micro-epidemiological scales in each population. Principal Component Analysis (PCA) was used to detect parasite sub-populations in each site, and tests of spatial autocorrelation including Moran’s *I* and spatial scan statistics were used to test for autocorrelation among parasite genotypes. The analyses were carried out at different spatial scales ranging from intensive within-village surveillance through to county-wide surveillance. 

## Materials and methods

### Study sites

*P. falciparum* infected blood samples were collected from individuals at three sites in two African countries: Kombo coastal districts of The Gambia on the West African coast; Kilifi, Kenya on the East African coast, and Rachuonyo South District in the Western Kenyan highlands. The Gambia has a subtropical climate with a single rainy season between the months of June and October ^33, 34^, while Kenya has two rainy seasons, experiencing short rains between October and December and long rains between April and August ^35^. In all three sites, *P. falciparum* is the main causative agent of malaria ^22, 33, 35^ and transmission occurs almost exclusively during and immediately after the rainy seasons ^34, 36^. The common vectors in The Gambia are *Anopheles gambiae s.s., Anopheles arabiensis* and *Anopheles melas* ^37^, while the common vectors in the Kenyan coast have historically been *A. gambiae s.s*. and *A. funestus*, but a recent shift to *A. arabiensis* and *A. merus* has been detected along the coast ^38^. In Rachuonyo South district, the main vectors transmitting malaria are *A. gambiae s.l*. and *A. funestus* ^39^. Temporal trends show declining malaria transmission in The Gambia and Coastal Kenya ^17, 33, 34, 40^, although not in Western Kenya ^41^. Asymptomatic parasite prevalence is lowest in The Gambia at 8.7% ^42^, intermediate in Kilifi at 14% ^43^ and slightly higher in Rachuonyo South at 16% ^44^. Over the study period, malaria transmission as measured by malaria slide positivity rate fell from 56% in 1998 to 7% in 2009 in Kilifi ^45^, and rose slightly in Fajara and Brikama in the Gambia ^33^.

### Ethics statement

Ethical approval for this study was obtained from Kenya Medical Research Institute (KEMRI) Ethical Review Committee (under SSC No. 2239). Written informed consent was obtained from parents/guardians of the study participants. The study methods were carried out in accordance with the approved guidelines.

### Sample collection, DNA extraction and Genotyping

5199 *P. falciparum* infected blood samples were collected during hospital admissions and community surveys over a 14-year period from 1998 to 2011. The Gambian samples were collected at Fajara and Brikama health facilities from children aged 8 months to 16 years who were living in the Kombo coastal districts and who were part of a clinical malaria study in 2007–2008 ^33^. The Kilifi samples came from children aged 1 to 6 years who had been recruited into a phase 2b randomized trial looking at the efficacy of the Candidate Malaria Vaccines FP9 ME-TRAP (multiple epitope–thrombospondin-related adhesion protein) and MVA ME-TRAP in 2005 ^46^, as well as clinical malaria studies looking at antibody responses to Merozoite Surface Protein 2 (MSP2) among individuals 3 weeks to 85 years old ^47^; the effect of declining transmission on mortality and morbidity in children up to 14 years old ^40^ and definitions of clinical malaria endpoints ^48^. The Rachuonyo south samples were collected during a community survey conducted in 2011 as part of a trial looking at the impact of hotspot targeted control interventions on reducing malaria transmission in the wider community ^22^. Prior to genotyping, DNA was extracted from these samples using either ABI prism 6100 Nucleic Acid prepstation (Applied Biosystems, Waltham, Massachusetts, USA) or Chelex Extraction.

276 SNPs in 177 genes were typed in the three parasite populations ( Dataset 1 ^49^). The SNPs were selected from a panel of 384 SNPs previously designed for a study on population structure of *P. falciparum* parasites from Africa, Southeast Asia and Oceania ^50^ and were chosen based on three criteria:

a) polymorphic among three of the most studied and well characterized *P. falciparum* strains (3D7, HB3 and IT).

b) uniformly distributed across the parasite genome.

c) ease of typing on the sequenom platform.

Genes typed included antigen-encoding, housekeeping and hypothetical genes. 52 and 9 SNPs were typed in the antigen-encoding parasite ligands Erythrocyte Binding Antigen 175 (EBA-175) and Apical Membrane Antigen 1 (AMA-1), respectively. In the Kilifi parasite population, between 158 and 226 SNPs were typed in each sample, while in The Gambia and Rachuonyo south populations, 131 and 111 SNPs were typed in 143 and 2744 samples, respectively. Genotyping was done on the Sequenom MassARRAY iPLEX platform, which allows multiplexing of up to 40 SNPs in a single reaction well and differentiates alleles based on variations in their mass ^51^. Locus specific PCR and iPLEX extension primers were designed with the sequenom MassARRAY designer software (Version 3.1) using 3D7 as the reference genome (PlasmoDB release 9.0) ( Dataset 2 ^52^). A multiplexed PCR reaction was performed by pooling locus-specific primers, and un-incorporated dNTPs were dephosphorylated enzymatically using shrimp alkaline phosphatase. Extension primers binding immediately adjacent to the SNP site of interest were then extended by a single nucleotide base, using mass-modified dideoxynucleotides. The extended products were resin cleaned to remove excess salts and the mass of the different alleles determined using MALDI-TOF mass spectrometry.

### Sample and SNP cut-off selection criteria

Genotype data was aggregated to determine genotyping success rates for individual samples and SNPs. Samples where >40% of SNP typing failed were excluded from analysis, and among the remaining samples, SNP typing for which >30% of samples failed were further excluded from analysis. The criteria for successful SNP typing were based on the SNP intensity values (r) and allelic intensity ratios (theta). Alleles were called as successful if they were above an intensity cut-off value ranging between 0.5 and 1.0, set depending on the performance of the individual SNP assay, and were classified as failed if they were below this cut-off. For those SNPs that were above the cut-off, allelic intensity ratios ranging between 0 and 1 were used to classify them as homozygous (single parasite genotype infections) or heterozygous (mixed parasite genotype infections). Theta values nearing 0 and 1 indicate different homozygous alleles, while intermediate values indicate heterozygous SNPs, representing mixed parasite populations. Where mixed parasite populations were identified, we took the majority SNP calls at each position to indicate the dominant genotype.

### Statistical analyses

All statistical analyses of genotype data were conducted in R statistical software (version 3.0.2) ^53^ except for the spatial scan statistics which were computed using SaTScan software (version 9.3) ^54^. Analyses were carried out separately for each parasite population, except for the Fixation index (FST) analyses which by definition involve the comparison of populations and so were carried out between samples in the different sites.

In each population, genotype data for all samples was aggregated and analysed collectively. Separate analyses were also carried out for subsets of SNPs typed in EBA 175 (39, 36 and 20 SNPs in The Gambia, Kilifi and Rachuonyo South, respectively) and AMA1 (9 SNPs in The Gambia and 8 SNPs in Kilifi). Only 3 SNPs were genotyped in Rachuonyo South, so this SNP subset was not analysed separately. In the Kilifi population, we ran additional analyses for samples collected from community surveys (asymptomatic infections) and hospital admissions (symptomatic infections).

***Calculating pairwise time, distance and SNP differences.*** Analyses were carried out separately for each of the three sites. Each parasite was compared to every other parasite in that site (i.e. a pairwise analysis), noting the time, distance and SNP differences between the parasite pair ( Dataset 3 – Dataset 5 ^55– 57^). We took half the lower limit of detection of temporal and spatial differences for parasite pairs collected on the same day and/or at the same location. Parasite pairs collected on the same day were assigned a difference of 0.5 days. For older samples in Kilifi (i.e. collected prior to 2004) where location was known to a 5 km accuracy, pairs collected at the same location were assigned a difference of 2.5km. We had precise geospatial co-ordinates for recent samples in Kilifi (i.e. collected after 2004) as well as all samples from The Gambia and Rachuonyo South, so parasite pairs in these three groups collected from the same location were assigned a difference of 0.02km.

SNP differences were computed by comparing genotype data for parasite pairs within each population and counting the number of SNPs between them. Missing SNP data for each parasite was replaced with the major allele in the respective population, after excluding SNP typing where >30% of assays failed as described above.

***Population genetics analyses.*** Minor allele frequencies were computed for SNPs in each population. Principal components analysis (PCA) was performed using singular value decomposition on a covariance matrix of pairwise SNP differences between parasites in individual populations. To detect inter-population genetic differentiation and within-population genetic diversity, we restricted analysis to 33 SNPs that had been successfully typed in all three populations.

***Spatial autocorrelation.*** Moran’s *I* was calculated using geographical coordinates to specify location and scores for the first 3 principal components to specify associated attribute values. Moran’s *I* was computed at distance classes of 1 km, 2 km and 5 km, using 100 bootstrap resampling steps to determine statistical significance.

Spatial scan statistics were calculated using SaTScan software and were run separately for each study site. The statistics involved running a purely spatial, retrospective analysis based on a normal probability distribution model using continuous variables (PC scores) and looking for areas with clusters of high PC scores. Latitude and longitude coordinates were used to represent the geographical locations of specific parasites, whereas principal component scores were used to represent individual parasite genotypes. During the analysis, a scanning window that gradually varies in size from including only a single homestead up to 50% of the population moves over the geographical space and at each window size and location, the ratio of parasites with high PCs inside the window versus outside the window is calculated. The window with the highest ratio is noted down as a cluster and its statistical significance is determined after accounting for multiple comparisons using random permutations.

***Raster analysis.*** To identify possible spatial barriers to parasite movement and mixing over short distances, each study area was divided into pixels of varying sizes which were then scored with 1 or 0, based on whether or not a straight line linking any two parasites crossed their boundaries. These pixels were then used as independent variables in a multivariable linear regression analysis that had the number of SNP differences as the dependent variable. Significance of the coefficient estimates were determined using non-parametric bootstrapping with 100 resampling steps.

To test for correlations between transmission intensity and population genetics at fine scale, each pixel was assigned the mean of the PC scores and either Malaria Positive Fraction (for Kilifi data) or asymptomatic parasite prevalence by PCR (for Rachuonyo) for all samples found within that pixel. The correlation between PC score and MPF or between PC score and parasite prevalence was tested by Spearman’s rank ordered correlation coefficient.

Dataset 1. Information on the 276 SNPs genotyped in 177 genes in P. falciparum parasite populations from The Gambia, Kilifi and Rachuonyo South The columns contain the following information: study_location, site of sample collection; sample_id, unique sample identifier; gene_symbol, gene name (if available); chr_valid, chromosome; coord_valid= base position of SNP on chromosome; sequence_code, SNP name; assay_code, name of assay; rsnumber, unique SNP identifier in dbSNP; reference_allele, 3D7 reference allele, alternative_allele, alternative allele; single letter code, IUPAC code for SNPs; result, genotype call after processing; allele1, IUPAC code for allele 1; allele2, IUPAC code for allele 2; allele_ratio1, proportion of allele 1; allele_ratio2, proportion of allele 2; pass_fail, coding of SNP based on availability of valid genotype (pass) or lack of a valid genotype (fail). Geospatial data for homestead location is considered sensitive data and therefore cannot be made open access. However, it can be accessed through a request to our data governance committee, using the email address mmunene@uat/newsite. Click here for additional data file. Copyright: © 2017 Omedo I et al. 2017 Data associated with the article are available under the terms of the Creative Commons Zero "No rights reserved" data waiver (CC0 1.0 Public domain dedication).

Dataset 2: Sequenom assay design information. Data includes the locus and IPLEX specific primers used in the sequenom reaction to amplify and type the SNPs of interest. Gene product, gene product name; Gene_symbol, gene name; Chromosome, chromosome location of gene; SNP position on chromosome, SNP site; reference allele, 3D7 reference allele; alternative allele, alternative allele; sequence, 3D7 reference sequence spanning the SNP site; first_pcrp, first PCR primer sequence; second_pcrp, second PCR primer sequence; extension_primer, IPLEX extension primer sequence; extension1_call, IPLEX primer with extended SNP;  extension1_mass, Mass of the extended IPLEX primer; extension1_sequence, sequence of extended IPLEX primer; extension2_call= IPLEX primer with alternative extended allele; extension2_mass, Mass of the extended IPLEX primer with alternative allele; extension2_sequence, sequence of extended IPLEX primer with alternative allele.  Click here for additional data file. Copyright: © 2017 Omedo I et al. 2017 Data associated with the article are available under the terms of the Creative Commons Zero "No rights reserved" data waiver (CC0 1.0 Public domain dedication).

Dataset 3: SNP, distance and time differences between P. falciparum parasite pairs in The Gambia population. Differences were computed for all parasite pairwise comparisons. Sample_id and sample_id_x are unique sample identifiers; snps represent the number of snp differences between parasite pairs; km_distance represents geographical distance, in kilometres, between parasite pairs; time_diff represents the temporal distance, in days, between parasite pairs. Click here for additional data file. Copyright: © 2017 Omedo I et al. 2017 Data associated with the article are available under the terms of the Creative Commons Zero "No rights reserved" data waiver (CC0 1.0 Public domain dedication).

Dataset 4: SNP, distance and time differences between P. falciparum parasite pairs in the Kilifi population. Differences were computed for all parasite pairwise comparisons. Sample_id and sample_id_x are unique sample identifiers; snps represent the number of snp differences between parasite pairs; km_distance represents geographical distance, in kilometres, between parasite pairs; time_diff represents the temporal distance, in days, between parasite pairs. Click here for additional data file. Copyright: © 2017 Omedo I et al. 2017 Data associated with the article are available under the terms of the Creative Commons Zero "No rights reserved" data waiver (CC0 1.0 Public domain dedication).

Dataset 5: SNP and distance differences between P. falciparum parasite pairs in the Rachuonyo South population. Differences were computed for all parasite pairwise comparisons. Sample_id and sample_id_x are unique sample identifiers; snps represent the number of snp differences between parasite pairs; km_distance represents geographical distance, in kilometres, between parasite pairs. Click here for additional data file. Copyright: © 2017 Omedo I et al. 2017 Data associated with the article are available under the terms of the Creative Commons Zero "No rights reserved" data waiver (CC0 1.0 Public domain dedication).

## Results

### Study populations

**Figure 1.**
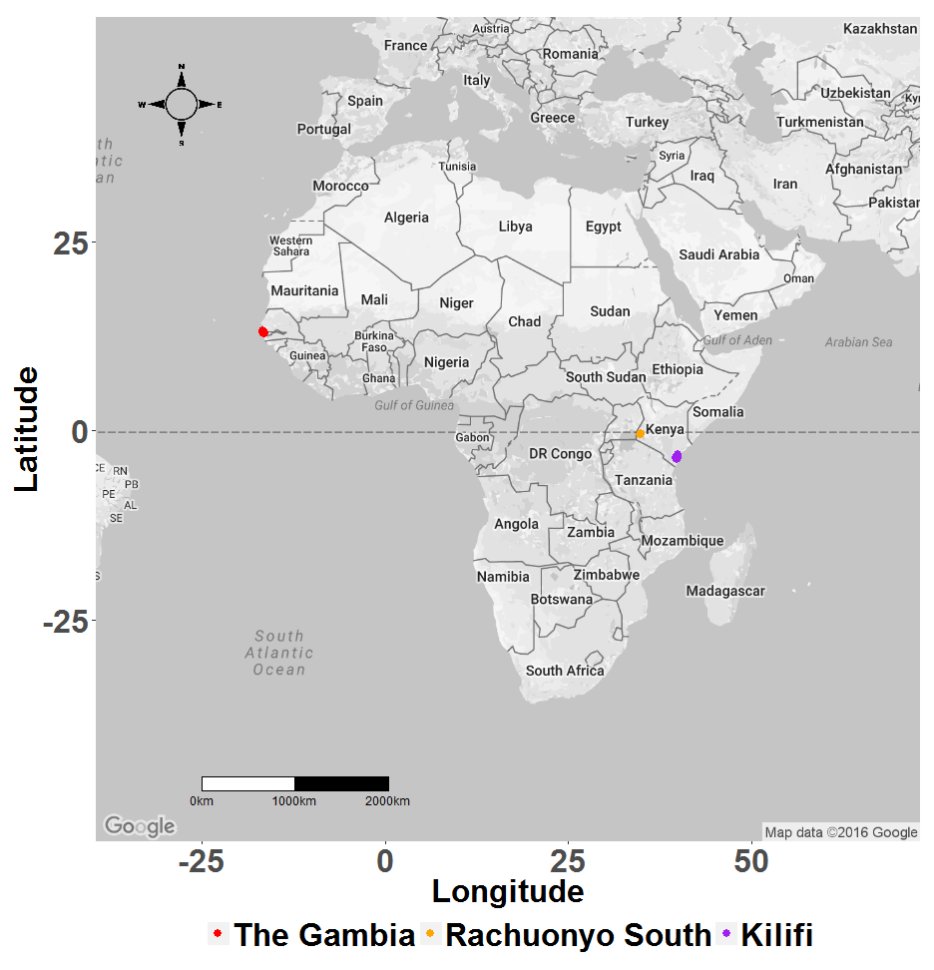
Map of Africa showing the three study sites. The study was conducted on *P. falciparum* samples collected in The Gambia, West Africa and Rachuonyo South District and Kilifi County in Kenya, East Africa.

5199 *P. falciparum* parasite isolates were collected from the Kombo coastal districts in The Gambia, and Kilifi County and Rachuonyo South district in Kenya ( Figure 1) between 1998 and 2011. 107, 177 and 82 SNPs were successfully genotyped in 133, 1602, and 1034 parasite isolates from The Gambia, Kilifi and Rachuonyo South district, respectively ( Table 1). 26, 57 and 49 SNPs were present at frequencies of 5% and above in The Gambia, Kilifi and Rachuonyo, respectively. In each of the populations, there was a positive correlation between SNP assay performance and parasite density.

**Table 1.** Summary of information on *P. falciparum* infected blood samples collected from The Gambia, Kilifi and Rachuonyo South study sites.

Study site	Contributing study	Study period	Average parasite density	Samples genotyped	Samples analysed	SNPs genotyped	SNPs analysed
**The Gambia ** **(Kombo ** **Coastal ** **Districts)**	Clinical malaria study	Sep ’07 – Dec ‘08	406,093	143	133	131	107
**Kilifi**	Community surveys	Feb – Oct ‘05	4562	748	195	240	177
**Kilifi**	Clinical malaria surveys	Jul ’98 – Apr ‘10	352,428	1564	1407	240	177
**Rachuonyo ** **South**	Community surveys	2011	NA	2744	1034	111	82

In all study sites, separate analyses of EBA175 and AMA1 did not reveal qualitatively different results from the pooled analyses ( Supplementary figure 1 – Supplementary figure 3) and only the results of the pooled analyses are presented here. In the Kilifi population, results were similar between the community surveys and hospital admissions. Here we present the results of the combined analyses of these data subsets.

### Parasite genetic diversity and population differentiation

Weir and Cockerham’s fixation index (F _ST_) estimates showed that the level of differentiation amongst the three populations was 0.046, comparable with results of other studies of African populations ^58, 59^. Pairwise population analysis gave F _ST_ values of 0.041 between Kilifi and Rachuonyo South, 0.078 between The Gambia and Kilifi and 0.108 between The Gambia and Rachuonyo South, showing the greatest genetic differentiation between The Gambia and Rachuonyo South parasite populations.

Analysis of within-population genetic diversity (π), based on a set of 33 SNPs that had been typed in samples from all three populations, showed that parasites in Rachuonyo South had the highest genetic diversity with an average of 3.384 (95% CI: 3.380 – 3.388) SNP differences per parasite pair. Those in The Gambia had the lowest SNP differences per parasite pair at an average of 2.867 (95% CI: 2.836 – 2.898) SNPs, while Kilifi had intermediate genetic diversity at 3.229 (95% CI: 3.226 – 3.231) SNP differences per parasite pair.

**Figure 2.**
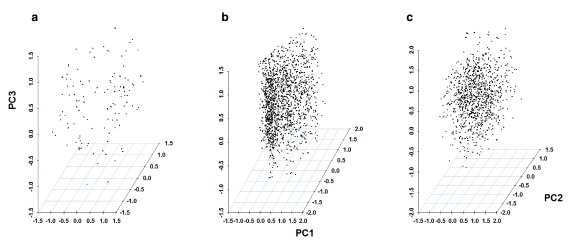
Plots of Principal Component Analysis scores for *P. falciparum* parasite populations in the study sites. Each point represents one of 133 parasites in The Gambia ( **a**), 1602 parasites in Kilifi ( **b**) and 1034 parasites in Rachuonyo South ( **c**). Genetic structuring was not observed for any of the parasite populations based on these three principal components. Cumulatively, the first three principle components accounted for 36.1% (PC1=18.4%, PC2=10.4%, PC3=7.3%), 13.2% (PC1=5.1%, PC2=4.4%, PC3=3.7%) and 12.7% (PC1=4.4%, PC2=4.3%, PC3=4%) of the variability seen in The Gambia, Kilifi and Rachuonyo South populations, respectively.

**Figure 3.**
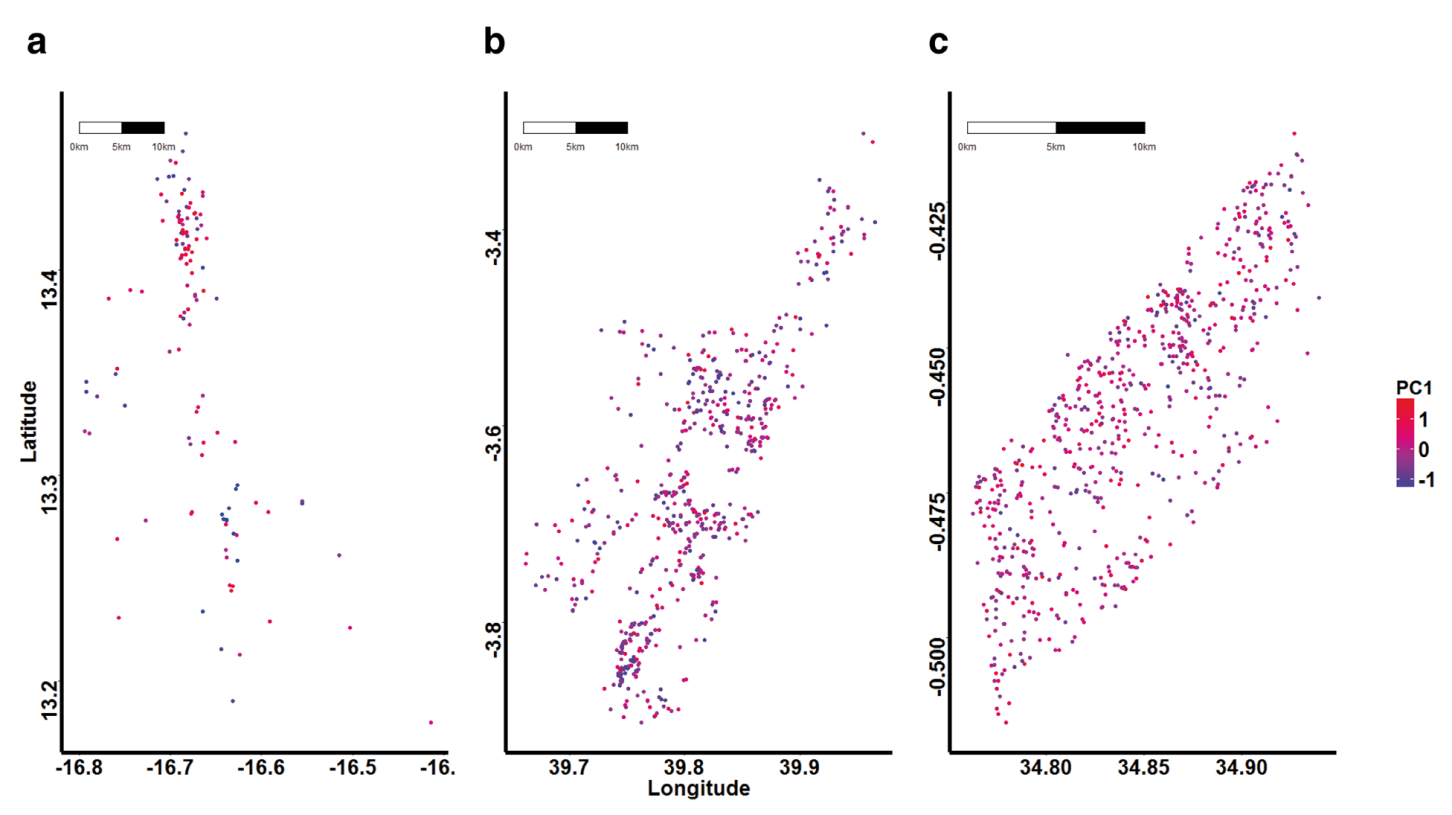
Geographic distribution of *P. falciparum* parasite genotypes based on scores for the first principal component. Each point represents the location of an individual parasite isolate and the colour shading represents distinct genotypes for parasites in ( **a**) The Gambia, ( **b**) Kilifi and ( **c**) Rachuonyo South study sites.

Principal Component Analysis (PCA) was carried out separately for each population using the 107, 177 and 82 SNPs that were successfully typed in The Gambian, Kilifi and Rachuonyo South parasite populations. Cumulatively, the first three principal components accounted for 36.1% (PC1=18.4%, PC2=10.4%, PC3=7.3%) of the variability seen in The Gambia, 13.2% (PC1=5.1%, PC2=4.4%, PC3=3.7%) of the variability seen in Kilifi and 12.7% (PC1=4.4%, PC2=4.3%, PC3=4%) of the variability seen in Rachuonyo South. We were unable to resolve parasite populations into distinct sub-populations using principal component analysis ( Figure 2 and Figure 3, Supplementary Figure 4).

### Global and local spatial autocorrelation analysis

**Figure 4.**
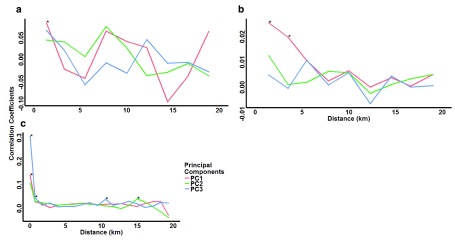
Moran’s *I* spatial autocorrelation analysis for the first three principal components. Coefficients were computed at distance classes of 2 km for ( **a**) The Gambia and ( **b**) Kilifi, and 1 km for ( **c**) Rachuonyo South parasite populations. Asterisks indicate distances at which parasites have significant (p<0.01) autocorrelations. In The Gambia and Kilifi populations, only a few samples were collected from the same location, so Moran’s *I* was not computed at this distance (0 km).

Having not seen sub-populations by PCA alone, we then included spatial analyses to test for spatial structure to the principal component values. Moran’s *I* analysis for spatial autocorrelation showed slight positive correlations for parasites that were statistically significant for at least one principal component at 2 km and below in The Gambia, 5 km and below in Kilifi, and 1 km and below in Rachuonyo South ( Figure 4).

Spatial scan statistics using SaTScan identified statistically significant (p≤0.01) clusters of distinct parasite sub-populations of different sizes in Kilifi and Rachuonyo South. In Kilifi, one cluster with a radius of 1.54 km (p=0.01) containing 15 parasites was detected, while in Rachuonyo South, a smaller cluster of genetically distinct parasites was detected with a radius of 0.5 km (p=0.001) containing 14 parasites. No clusters were detected in The Gambian population, indicating that parasites did not group into distinct sub-populations in this study site.

### Spatio-temporal variations in genetic differences between parasite isolates

**Figure 5.**
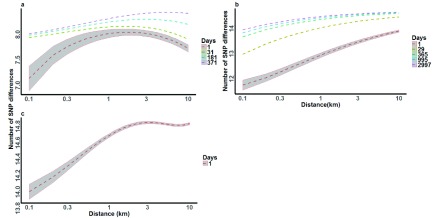
Effects of time-distance interaction on the number of SNP differences between parasite pairs. Dashed lines represent time intervals separating parasite pairs in ( **a**) The Gambia, ( **b**) Kilifi and ( **c**) Rachuonyo South study sites. 95% confidence intervals are included around the 1-day curves in each study site. 100 pairwise analyses were used to generate the curves at each time point. 107, 177 and 82 SNPs were analysed in The Gambia, Kilifi and Rachuonyo South parasite populations, respectively. Dummy data used to generate the graphs contained 8 SNPs in the Gambia, 14 SNPs in Kilifi and 10 SNPs in Rachuonyo south.

We examined the effect of distance and time separating parasite pairs on genetic relatedness to determine the spatial extent and rate of parasite mixing. We used linear regression models where the number of SNP differences between parasite pairs was an outcome predicted by the distance between parasite pairs and the time between parasite pairs. Time was not included for the Rachuonyo South population as the samples were collected in a single cross-sectional survey taken over a few days. Across all three datasets, distance was independently associated with increasing variation in genotype, i.e. the further apart in space any two parasites were, the greater the number of SNP differences between them. In The Gambia and Kilifi populations, time was also shown to be associated with increasing variation in genotype, with parasite pairs collected further apart in time having greater number of genetic differences. Additionally, in The Gambia and Kilifi populations, time interacted antagonistically with distance to attenuate the effect of distance on genotype relatedness ( Figure 5). This means that the genetic differences between any two parasites increased with distance, but at a decreasing rate when time between these samples increased. We observed that in The Gambian population, parasites acquired SNP differences over distance at a slower rate than in the Kilifi and Rachuonyo populations.

Bootstrapping the analyses (to take into account the linked nature of pairwise observations) gave statistically significant effects of distance, time and the interaction between distance and time ( Table 2).

**Table 2.** 95% bootstrap confidence intervals for the linear effects of time, distance and the interaction of time and distance on changes in SNP differences between parasite pairs.

	Time (days)	Distance (km)	Time-Distance interaction
**The Gambia**	-0.005 - -0.001 (p=0.004)	0.086 – 0.723 (p<0.001)	-0.0003 - -0.002 (p=0.003)
**Kilifi**	0.190 – 0.647 (p<0.001)	0.297 – 1.363 (p=0.001)	-0.453 - -0.072 (p=0.003)
**Rachuonyo South**	-	0.0104 – 0.275 (p=0.018)	-

Values represent the change in the number of SNP differences between parasite pairs per day (time), per kilometre (distance) and per day/kilometre (time-distance interaction). Time, distance and the product of time and distance (time-distance interaction) were log transformed prior to running the regression analyses.

### Identification of geographical barriers to parasite movement

We conducted raster analysis by pixels to examine a) the spatial relationship between distinct parasite genotypes as represented by the principal component analysis and either malaria positive fraction (MPF) data (in Kilifi) or PCR positive data (in Rachuonyo South) and b) the presence of possible spatial barriers to parasite movement that would act as factors. “The range of MPF and parasite prevalence per pixel varied depending on the size of the pixels analysed. In the Kilifi population, MPF ranged from 0 – 100% (interquartile range (IQR) = 20%) for the 0.5km pixels; 0 – 100% (IQR = 14%) for the 1.0km pixels; 20 – 83% (IQR = 7%) for the 2km pixels; and 33 – 63% (IQR = 4.7%) for the 4km pixels. In the Rachuonyo South population, PCR positive prevalence varied from 0 – 75% (IQR = 19.4%) for the 0.5km pixels; 0 – 47% (IQR = 17.4%) for the 1.0km pixels; 3.5 – 35.8% (IQR = 14.3%) for the 2km pixels; and 6.2 – 33.4% (IQR = 7.8%) for the 4km pixels.”

The analysis of principal components did not show any consistent or statistically strong associations with markers of transmission intensity (i.e. malaria positive fraction and prevalence of asymptomatic parasitaemia by PCR) ( Supplementary Figure 5).

Bootstrapping the multivariable linear regression analysis of pairwise comparisons of samples for SNP differences using 189, 703 and 340 pixels for The Gambia, Kilifi and Rachuonyo South, respectively, showed that the majority of pixels were not significant influences on SNP differences ( Supplementary Figure 6). The few pixels that were significant (p<0.05) were non-significant after applying Bonferroni correction to account for multiple testing. Furthermore the distribution of p values was uniform for each dataset (mean p value ~0.5 in each population).

## Discussion

As malaria transmission declines, targeted control at the micro-epidemiological scale is likely to be important in eliminating malaria in any remaining transmission foci. The effectiveness of such targeted measures will depend on the extent of parasite mixing in and around these foci ^23^. In the current analysis, we did not identify any population structure by simple inspection of the Principal components derived from SNP genotyping in The Gambia, Kilifi and Rachuonyo South ( Figure 2 and Figure 3), indicative of a parasite population that is well mixed. However we did not conclude that there was no structure to the population, only that we could not identify it in the absence of spatial data. We therefore went on to analyse the genotype data using spatio-temporal data, and identified spatial autocorrelation using Moran’s *I* in all three populations, with statistical significance (p<0.01) for the first principal component in The Gambia and Kilifi and the third principal component in Rachuonyo South ( Figure 4). Overall, the consistent pattern observed in the Moran’s *I* analyses was that of spatial auto-correlation at close proximity (i.e. at a range of a few km), and little or no auto-correlation at larger distances. The auto-correlation was modest in effect size but statistically significant with p values ranging from 0.01 to 0.001 at < 1 km. However, using scan statistics we identified only two specific clusters of distinct parasite sub-populations based on PC scores, one in Kilifi and another in Rachuonyo South. The limited evidence of specific local clusters of parasite sub-populations in the face of evidence of spatial auto-correlation over the whole study site implies that there is a high degree of mixing among parasites within the study sites, leading to limited clustering of parasites into genetically distinct sub-populations.

We further looked at the effect of time, distance and time-distance interaction on the variation in SNP differences between parasite pairs within individual study sites. Since the number of days differed for almost all parasite pairs, dummy data were included in the regression analysis to enable the generation of time-distance interaction graphs. For each study site, a distance range of 1 – 10km (with an interval of 0.1km between adjacent distances) was used. Temporal distance with 14 and 10 day intervals were assigned to parasite pairs in Kilifi and the Gambia, respectively, whereas time was not considered for the Rachuonyo South population. Constant SNP differences of 14, 10 and 8 were used for parasite pairs in Kilifi, Rachuonyo South and the Gambia, respectively. We found that time and distance were independently associated with increasing variation between parasite genotypes (i.e. the further apart in time or space two parasites were, the greater the genetic differences observed between them). However, in the case of The Gambia and Kilifi populations where we had longitudinal data, time was shown to interact antagonistically with distance, with an increase in time reducing the variations in genetic differences between parasites as distance between the parasites increased ( Figure 5). This implies that distance between samples was no longer predictive of genetic variation when there were longer time periods between samples, indicating that, given enough time, even parasites that are separated by large distances would get a chance to interact and recombine, especially if they are not geographically isolated. The number of SNP differences were seen to plateau at approximately 1km in the Gambia, 3km in Kilifi and 10km in Rachuonyo South. This may be attributed to the characteristics of the local parasite population, which in turn may be explained by the distribution of human settlement in the areas sampled, for example in the Gambia, homesteads tend to be clustered together in distinct, autonomous villages whereas in Rachuonyo South there is a denser and more uniform pattern of human settlement over the study area, enabling the interaction of parasites over a much larger distance.

Lack of genetic structuring of parasite populations observed in this study is indicative of a population that is well mixed. This observation of a highly mixing parasite population is in agreement with results of similar studies of parasite genetic diversity and population differentiation using microsatellites ^58, 60, 61^, immune selected genes ^62, 63^ and SNPs ^64^. These studies were carried out in parasite populations from different geographical regions representing a diverse range of transmission intensities, from the highest in Africa and oceania, intermediate in Southeast Asia, to the lowest in south America. However, other studies have shown population structure when looking at the same population ^50, 65– 67^, although these analyses were carried out on larger geographical scales than those analysed here and mostly involved analyses at provincial, country or continental levels. Population structure was most evident in regions with low transmissiion intensities such as south America or southeast Asia ^58^, and less evident in Africa where transmission intensity is much higher ^61^.

On an international level, for example, some studies have been able to distinguish between Senegalese and Thai parasite isolates using a 24-SNP barcode ^68^, and another study using 4 SNPs out of a set of 384 SNPs was able to resolve East and West African parasites ^50^, showing that parasite populations can be resolved on a large geographical scale. A study in Senegal was also able to identify population structure among parasites using a 24 SNP barcode, despite a high level of similarity among the parasites analysed ^69^. It is possible that more detailed genotyping using a larger number of markers, for instance by whole genome sequencing, would start to identify mutations that are private to particular sub-populations at a finer geographical scale, although the degree of mixing observed here suggests that discrete populations are unlikely.

We identified spatial autocorrelation among parasites in the different study areas. However, most of these correlations were found over short distances, pointing to the existence of parasite sub-populations over small spatial scales. This indicates the presence of clusters of genetically distinct parasites at micro-epidemiological scales within the study sites. Previous studies have identified parasite sub-populations based on clustering of serological responses to the important antigen *Plasmodium falciparum* Erythrocyte Membrane Protein 1 ( *Pf*EMP1) in children in Kilifi ^70^, supporting our observations of parasite sub-populations at this site. In Papua New Guinea, sub-populations of parasites have also been identified at a micro-epidemiological scale using PfEMP1 ^71^, indicating that this may be a good marker for population differentiation at the micro-epidemiological level.

Studies on hotspots of symptomatic malaria infection have identified hotspots or clusters of infections down to the level of individual homesteads in Kilifi ^9^. The lack of consistent correlations between parasite genotypes and infection prevalence shown through raster analysis of pixels in this study ( Supplementary Figure 5) indicate that infections within higher incidence areas are likely not caused by distinct parasite sub-populations. Instead, such infections are likely caused by parasites that are well mixed within the general population. Our inability to detect barriers to parasite movement over short distances indicates that parasites move freely within the study areas, and the spatial extent of such parasites may be limited only by the ecology and dispersal range of mosquito vectors. Furthermore, recent examination of the epidemiology of hotspots shows that they occur at the full range of spatial scales, with a pattern of spatial auto-correlation that does not show a discontinuity at any scale (i.e. a smooth semi-variogram) ^9^. This further argues against the existence of discrete “units” of transmission with sub-populations of parasites.

This has implications for public health interventions that may target transmission hotspots. If hotspots consist of distinct parasite populations that do not mix with parasite populations in the wider parasite community, the impact of hotspot-targeted interventions beyond the hotspot boundaries can be expected to be limited. If parasites mix freely, as suggested by our data, the impact of hotspot-targeted interventions may affect community-wide malaria transmission. This assumes that hotspots can be detected, are stable in time ^20^ and the spread of parasite populations indeed primarily occurs from hotspots to the surrounding community ^23^.

This study had some limitations. First, the number of SNPs typed was relatively small, and this would have limited our power to detect genetic structuring among the highly similar parasite populations, especially in The Gambia. Detecting structuring in highly similar parasite populations may require either a much larger panel of SNPs or the use of more informative SNPs, as shown in the study by Campino *et al*, 2011 ^50^. Advances in sequencing technologies have increased the use of whole genome sequence data in the analysis of *P. falciparum* parasite population genetics, and this has led to the identification of hundreds of thousands of SNPs, most of which are present at very low frequencies especially in African parasite populations ^72^. Additional analyses will require the use of whole genome sequence data to identify rare variants and distinguish between closely related parasites, thus allowing parasite population structure to be analysed at fine spatial scales. However, despite the small SNP panel used in this study, we were still able to detect population structuring on a micro-epidemiological scale. Our analysis suggests that this structure was a uniform spatial and temporal auto-correlation rather than driven by discrete clusters of parasites at specific locations. Despite the limitations of our SNP typing and sample size we can therefore conclude that any specific clustering is less prominent as a feature than the auto-correlations in space and time that we can detect. 

A second limitation is that we conducted our study in only two sites in Kenya, and one site in the Gambia. It may be premature to generalize our results more widely and an analysis of more sites will be required to make confident generalizations. On the other hand the three sites selected do demonstrate differing transmission intensities typical of many endemic Sub Saharan African countries, and this was reflected in the level of genetic diversity observed in the populations. Furthermore, our findings are consistent across all three sites. Nevertheless, patterns of parasite mixing may differ between populations based on distinctive features such as geographic isolation and patterns of human movement. Further data are required to make more general conclusions. Furthermore, as transmission continues to decline and malaria programmes gradually shift their focus from control to elimination, the analysis of parasite gene flow between different transmission foci, e.g. Kilifi and Rachuonyo South, will become increasingly important in informing the mitigation measures needed to prevent importation of parasites as a result of human movement and migration. These analyses were not carried out in the current study since the numbers of common SNPs between the two Kenyan sites was low, and we only had parasites from one timepoint in Rachuonyo South, hence we were unable to conduct an informative analysis of gene flow between sites.

Finally, we used genetic data to show that there is high parasite movement and mixing within individual study sites. Additional analyses using gene flow models, e.g. as implemented in Migrate-N software, can be used to further validate our hypothesis of rapid gene flow and to confirm whether the parasites are part of a panmictic population or whether there exists underlying population structure, as well as to determine directionality of parasite movement between different populations, assuming that such populations can be identified within the region.

In conclusion, we have shown that *Plasmodium falciparum* parasite populations mix evenly within The Gambia, Kilifi and Rachuonyo South and there appear to be no detectable geographical barriers to parasite movement over short distances within these sites. That said, autocorrelations of genotype were detected at the micro-epidemiological level. We would conclude that control strategies that efficiently target hotspots will likely benefit the wider community outside the hotspots at the District/County level (we are however unable to comment on larger geographical scales), although this is likely to be affected by factors such as the underlying transmission level, heterogeneity of transmission, and patterns of human movement ^23^. On the other hand, based on the high level of parasite mixing observed at each study site, we would predict that ineffective application of control interventions such as mass drug administration that result in residual foci of transmission would lead to rapid re-infection of the wider community, and also that parasites acquiring mutations conferring drug resistance or immunological escape would spread rapidly at the micro-epidemiological level. This underscores the need for effective and sustained control until malaria elimination is achieved.

## Data availability

The data referenced by this article are under copyright with the following copyright statement: Copyright: © 2017 Omedo I et al.

Data associated with the article are available under the terms of the Creative Commons Zero "No rights reserved" data waiver (CC0 1.0 Public domain dedication). 

Figshare: Dataset 1: Information on the 276 SNPs genotyped in 177 genes in *P. falciparum* parasite populations from The Gambia, Kilifi and Rachuonyo South, doi: https://dx.doi.org/10.6084/m9.figshare.5383969 ^49^

Figshare: Dataset 2: Sequenom assay design information, doi: http://dx.doi.org/10.6084/m9.figshare.4640719 ^52^

Figshare: Dataset 3: SNP, distance and time differences between *P. falciparum* parasite pairs in The Gambia population, doi: http://dx.doi.org/10.6084/m9.figshare.4640722 ^55^

Figshare: Dataset 4: SNP, distance and time differences between *P. falciparum* parasite pairs in the Kilifi population, doi: http://dx.doi.org/10.6084/m9.figshare.4640725 ^56^

Figshare: Dataset 5: SNP and distance differences between *P. falciparum* parasite pairs in the Rachuonyo South populationm, doi: http://dx.doi.org/10.6084/m9.figshare.4640728 ^57^

## Supplementary material

**Supplementary figure 1.** Time-distance interaction curves showing the effect of distance on changes in genetic variation between Gambian *P. falciparum* parasite pairs over time. The analyses were carried out for **a**) pooled SNPs, **b**) EBA-175, **c**) AMA1 and **d**) ‘other’ SNPs (all SNPs excluding EBA175 and AMA1). Dashed lines represent time intervals separating parasite pairs at 1 day (red), 1 month (green), 6 months (blue) and 1 year (purple). The interaction term was log-transformed prior to running the analysis.

**Supplementary figure 2.** Time-distance interaction curves showing the effect of distance on changes in genetic variation between Kilifi *P. falciparum parasite* pairs over time. The analyses were carried out for **a**) pooled SNPs, **b**) EBA-175, **c**) AMA1 and **d**) ‘other’ SNPs (all SNPs excluding EBA175 and AMA1). Dashed lines represent time intervals separating parasite pairs at 1 day (red), 1 month (green), 6 months (blue) and 1 year (purple). The interaction term was log-transformed prior to running the analysis.

**Supplementary figure 3.** Time-distance interaction curves showing the effect of distance on changes in genetic variation between Rachuonyo South *P. falciparum* parasite pairs over time. The analyses were carried out for **a**) pooled SNPs and **b**) EBA-175. Parasites were all collected during a single cross sectional study thus time was not considered.

**Supplementary Figure 4. Geographical distribution of *P. falciparum* parasite genotypes based on scores for the second (PC2) and third (PC3) principal components.** Each point represents an individual parasite isolate and the colour shading represents distinct genotypes for parasites in The Gambia ( **d** and **g**), Kilifi ( **e** and **h**), and Rachuonyo South ( **f** and **i**) study sites.

**Supplementary Figure 5. Raster analysis by pixels.** This was carried out to determine the spatial relationship between distinct parasite genotypes as represented by principal component analysis and either malaria positive fraction (MPF) or PCR positive fraction (PPF) data. ( **a**) and ( **b**) show the distribution of scores for the first principal component (PC1) and MPF over a 1 km × 1 km grid area of Kilifi. ( **d**) and ( **e**) show the distribution of scores for the first principal component and PPF over a 1 km × 1 km grid area of Rachuonyo South. Spearman’s correlation coefficients computed to show the relationship between parasite genotypes and either MPF ( **c**) or PPF ( **f**) showed no strong associations between genotypes and the two markers of transmission.

**Supplementary Figure 6. Raster analysis by pixels to examine the presence of spatial barriers to parasite movement.** The pixel plots represent p values of bootstrapped linear regression correlation coefficients and show the significance of different geographical locations in acting as barriers to parasite mixing. Individual grid sizes were of approximately 1 km × 1 km in ( **a**) Kilifi and ( **c**) The Gambia and 0.5 km × 0.5 km in ( **b**) Rachuonyo South. The colour key in each case indicates the range of p values from 0.0001 to 1. Significant p values shown on the plot were non-significant after applying Bonferroni correction to account for multiple testing.
