# Supplementary figures and images for: Micro-epidemiological structuring of Plasmodium falciparum parasite populations in regions with varying transmission intensities in Africa
Source: Wellcome Open Res. 2017 Sep 8;2:10. Originally published 2017 Feb 14. [Version 2] doi: 10.12688/wellcomeopenres.10784.2 (PMC5445974; doi:10.12688/wellcomeopenres.10784.2)

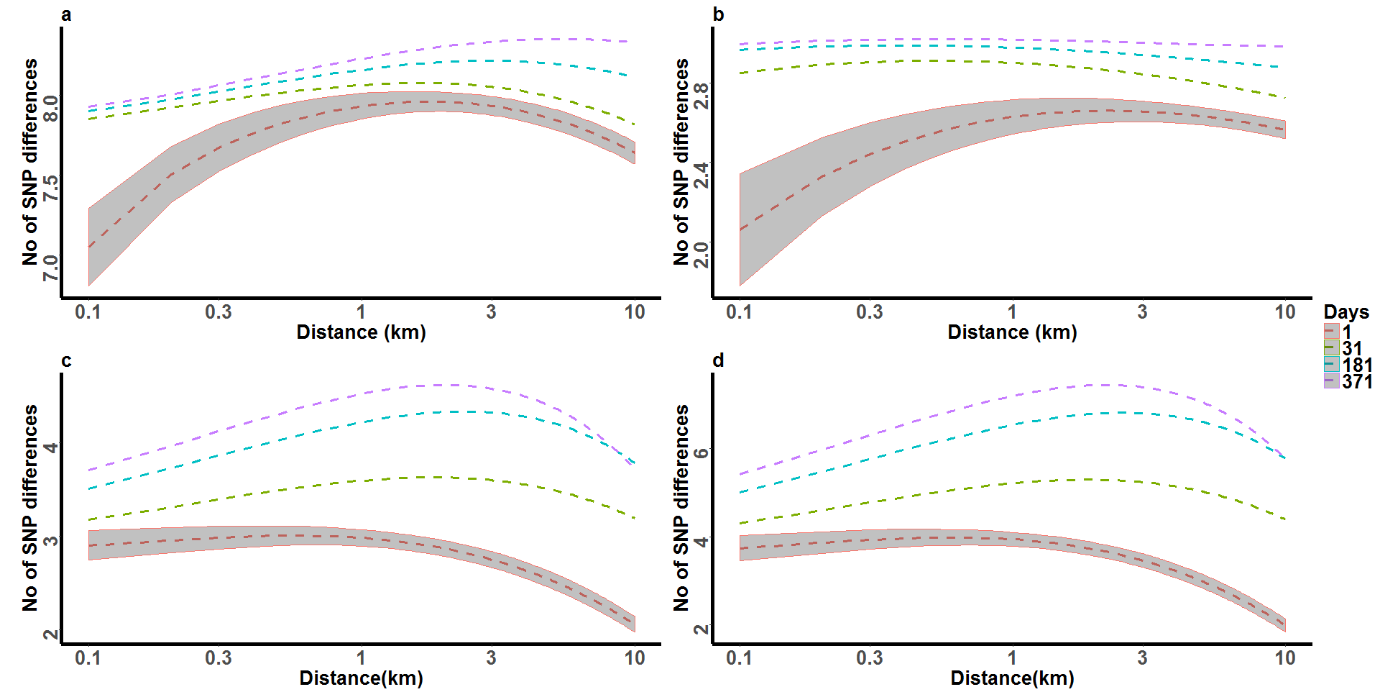

Supplement: Supplementary file 6 [file wellcomeopenres-2-13603-s0008.tgz › 03917ed1-b028-4a2c-ac51-e575cf313fb5.tif]

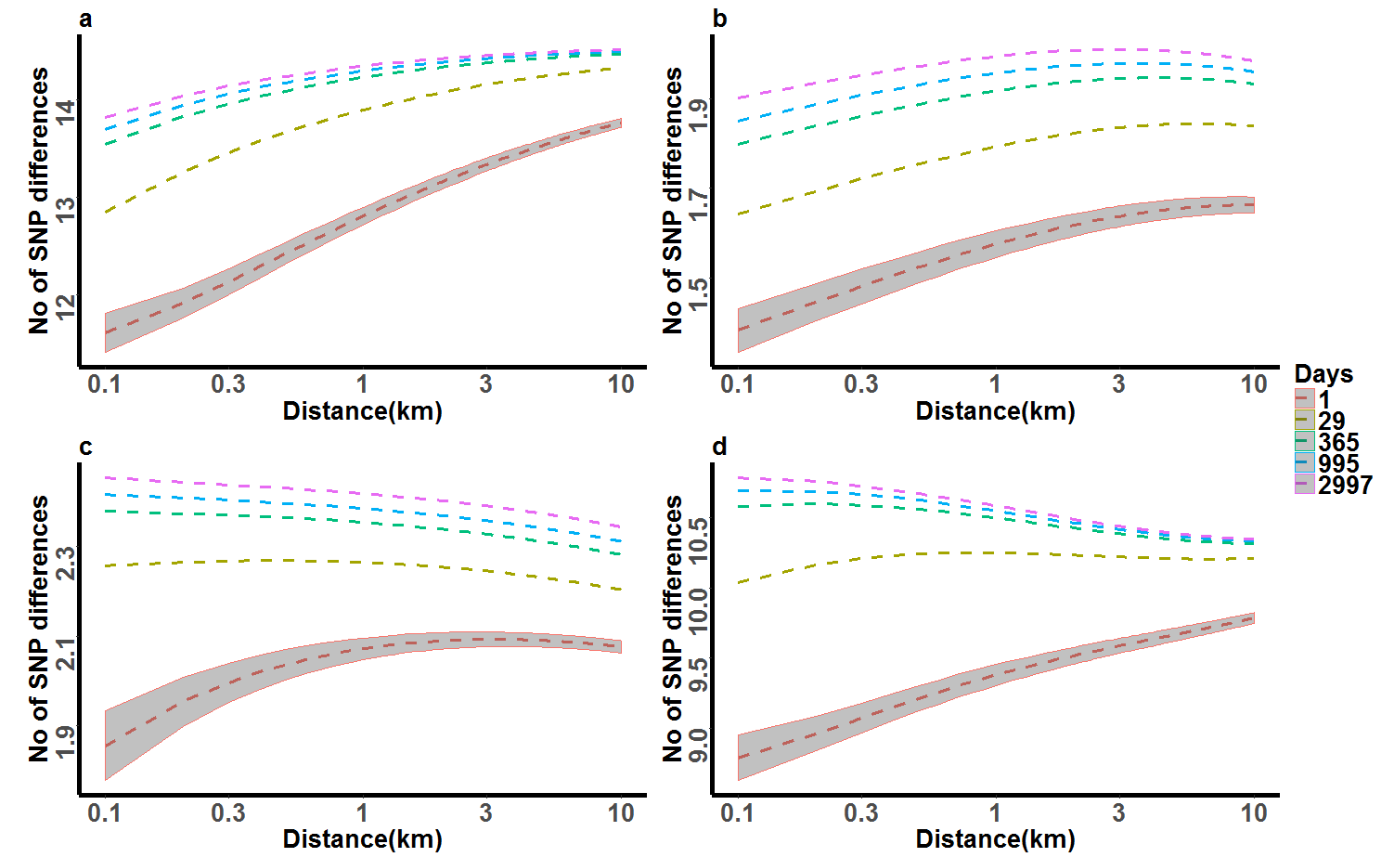

Supplement: Supplementary file 7 [file wellcomeopenres-2-13603-s0009.tgz › cd65c9f6-10b8-4ca0-834f-f67fd0b4ae4d.tif]

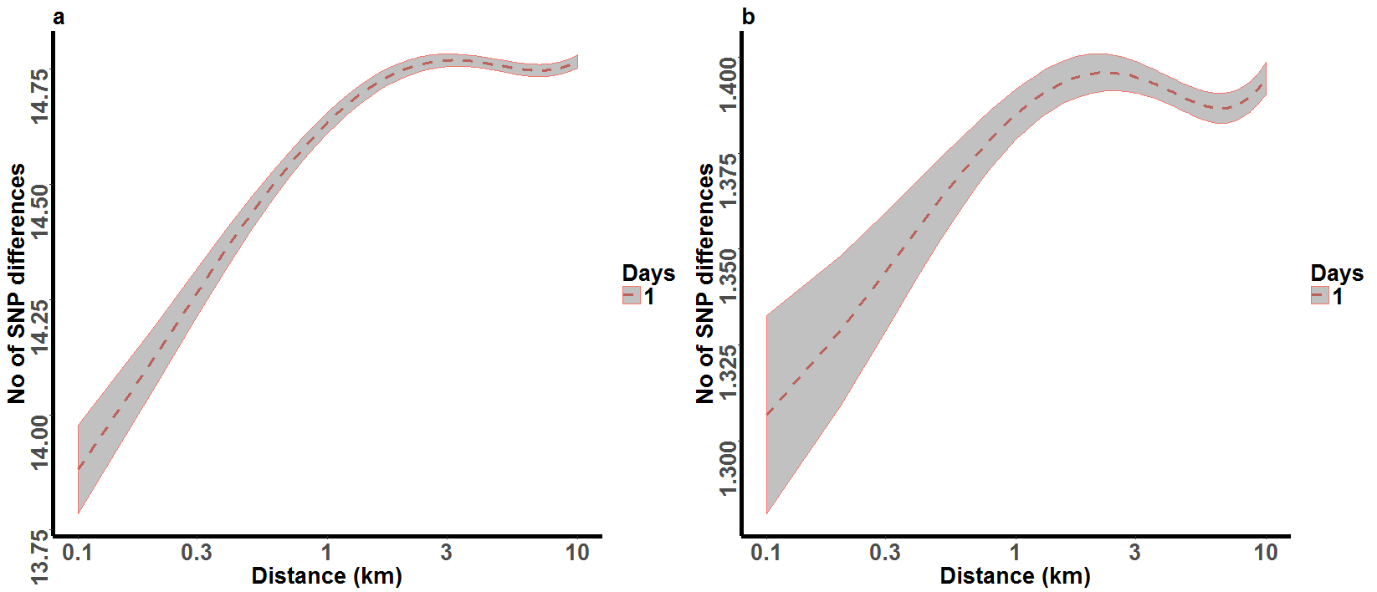

Supplement: Supplementary file 8 [file wellcomeopenres-2-13603-s0010.tgz › 3e9919ae-8693-49fb-91c4-f68566d063f5.tif]

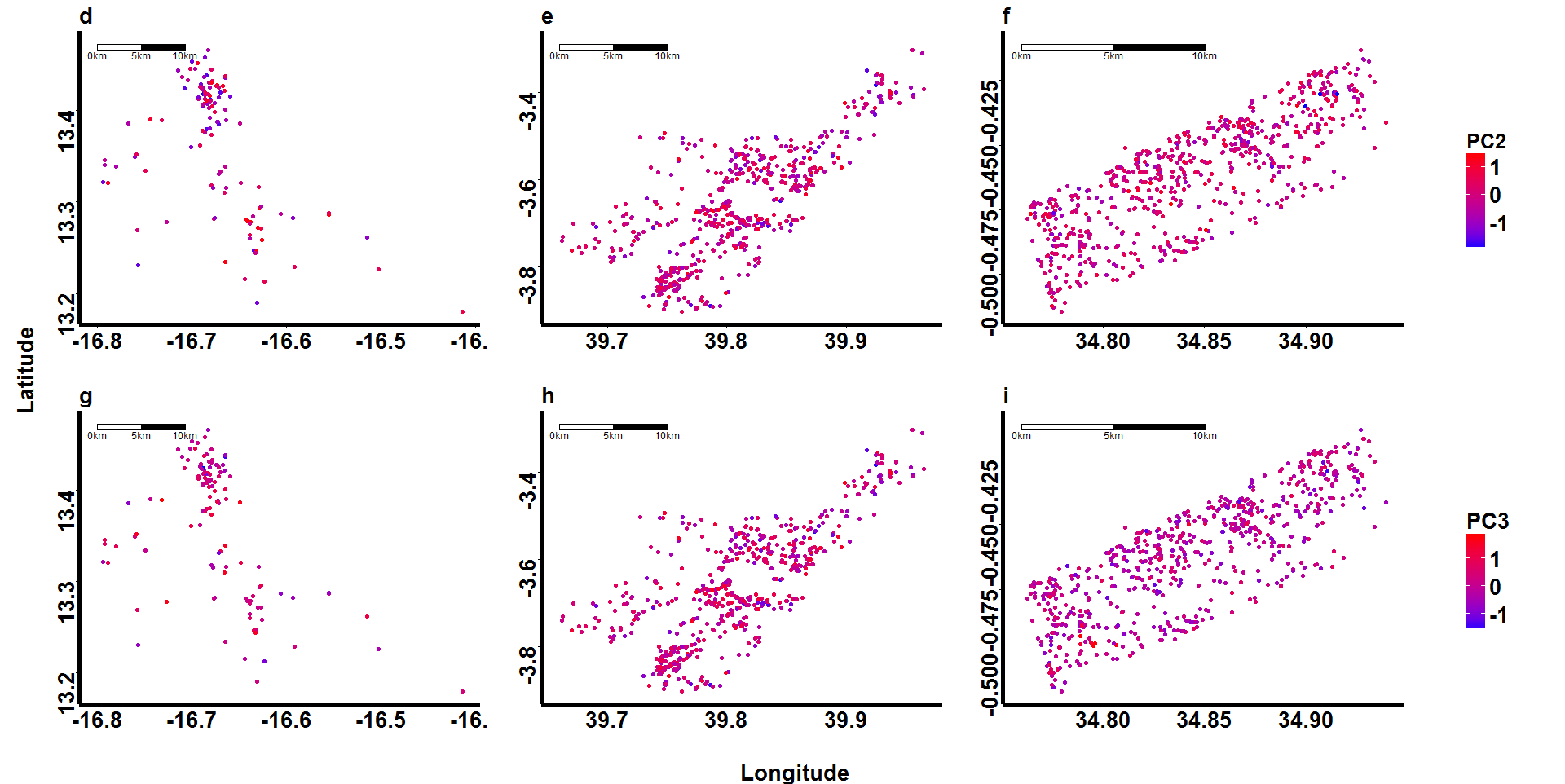

Supplement: Supplementary file 9 [file wellcomeopenres-2-13603-s0005.tgz › 908b4fca-9d84-44ba-842b-de34e73b7e63.tiff]

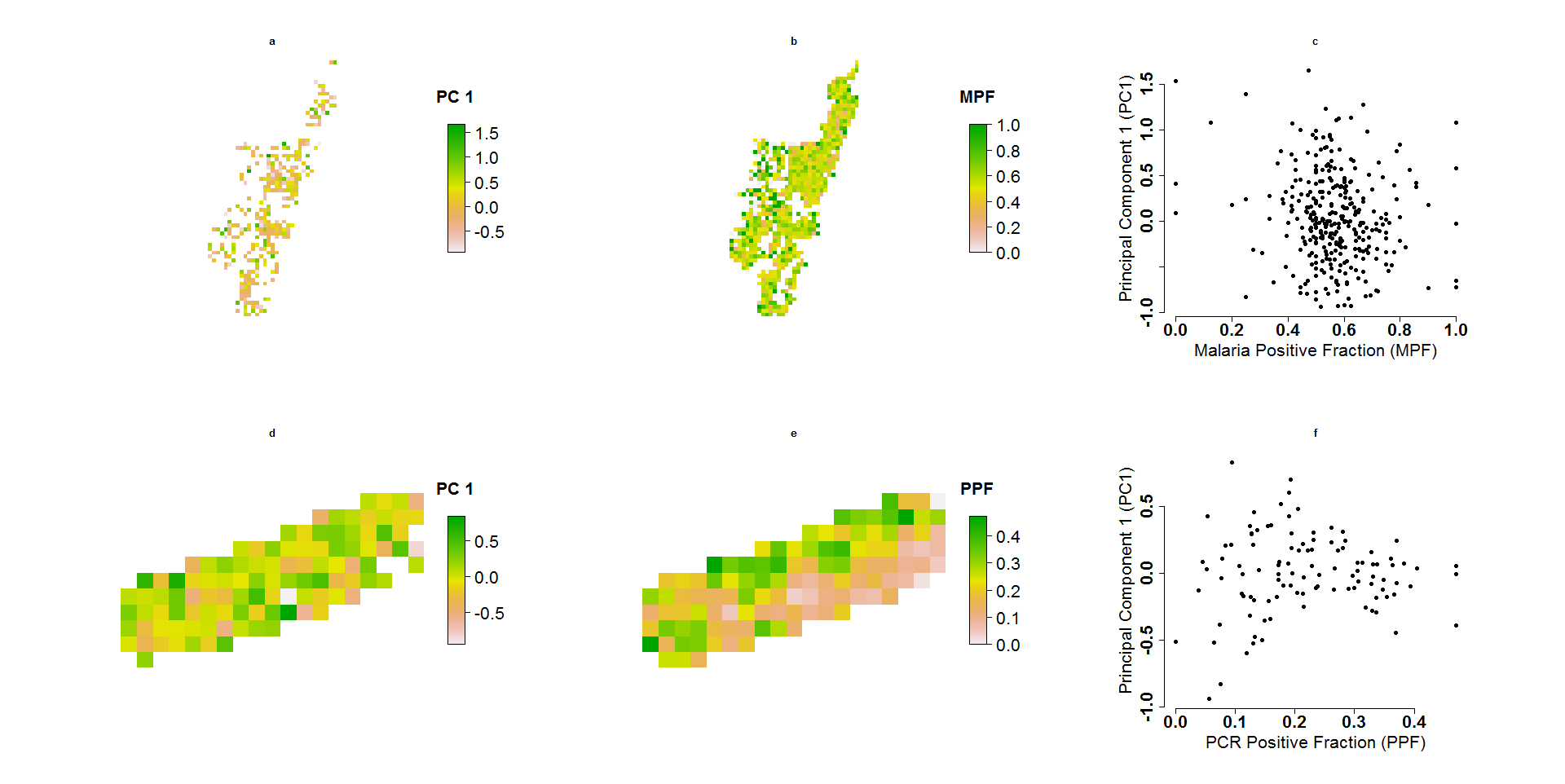

Supplement: Supplementary file 10 [file wellcomeopenres-2-13603-s0006.tgz › 0ce6512a-d070-461b-aed2-9cd4ca627630.tiff]

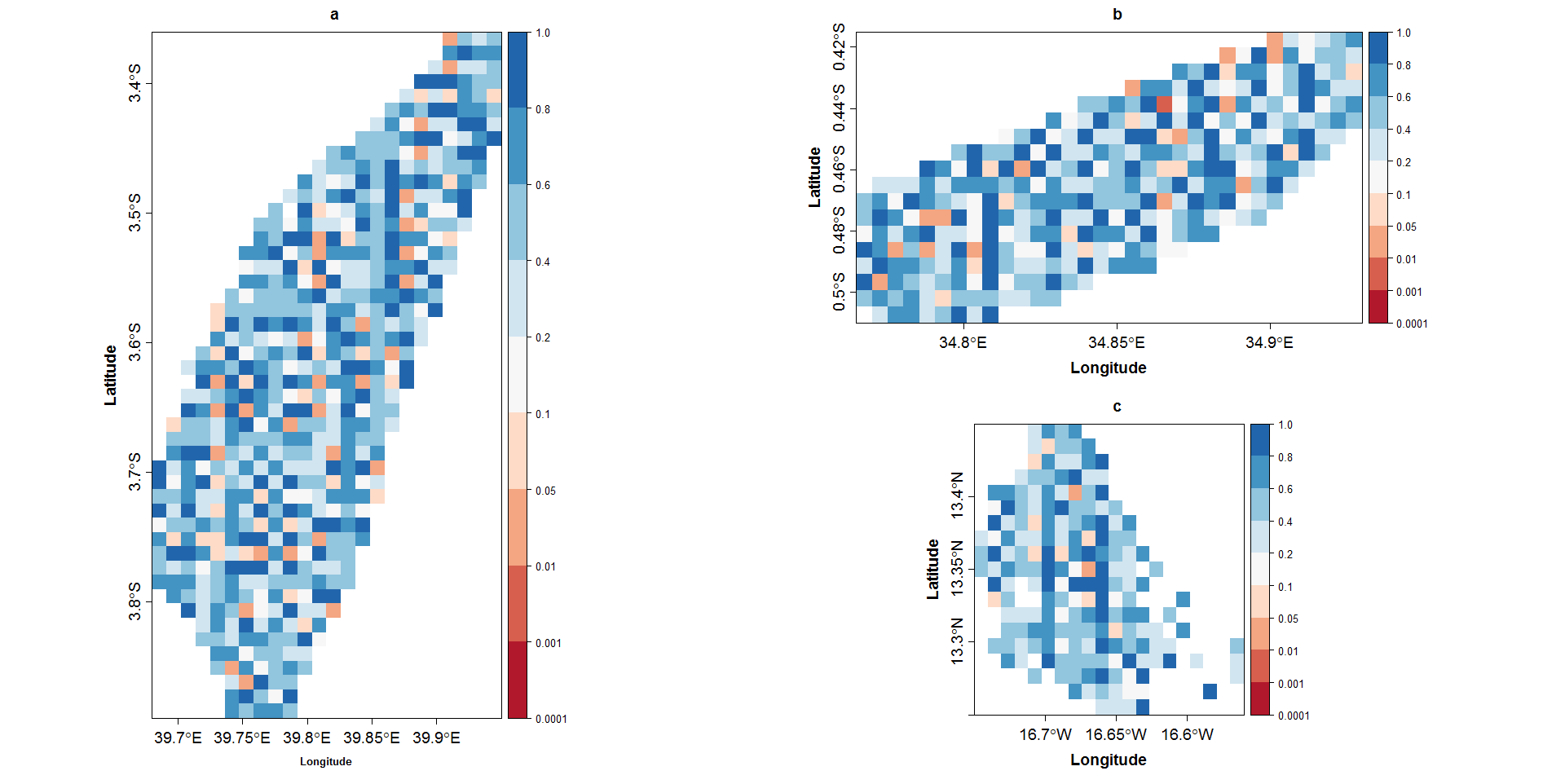

Supplement: Supplementary file 11 [file wellcomeopenres-2-13603-s0007.tgz › 3cdf03cf-3164-4913-92a7-f7df24336acc.tiff]
