# Supplementary material for: Micro-epidemiological structuring of Plasmodium falciparum parasite populations in regions with varying transmission intensities in Africa
Source: Wellcome Open Res. 2017 Sep 8;2:10. Originally published 2017 Feb 14. [Version 2] doi: 10.12688/wellcomeopenres.10784.2 (PMC5445974; doi:10.12688/wellcomeopenres.10784.2)
Supplement: Peer review history

# Peer review: Referee response for version 2

**DOI:** 10.21956/wellcomeopenres.13603.r25837

**Published:** 2017-09-08

**Authors:** Christopher Delgado-Ratto

**Affiliations:** 1 Global Health Institute, University of Antwerp, Antwerp, Belgium

I acknowledge the answers to my comments by the authors. I have no further comments on this new version.

I have read this submission. I believe that I have an appropriate level of expertise to confirm that it is of an acceptable scientific standard.

# Peer review: Referee response for version 1

**DOI:** 10.21956/wellcomeopenres.11628.r23565

**Published:** 2017-06-19

**Authors:** Liwang Cui

**Affiliations:** 1 Department of Entomology, Pennsylvania State University, State College, PA, USA

This study analyzed large sample sets of malaria parasites taken from the western and eastern coasts of Africa (The Gambia and Kenya) and genotyped at 276 SNPs. For two of the sample sets, parasites were collected at different time points, allowing identification of population changes over time and space. Overall, the analysis was sound and results were well explained. The authors also notified the limitations of the study. For example, inclusion of additional parasite samples between these western and eastern sites, and use of more SNP markers would validate whether the conclusions drawn here represent the whole African continent.

 Comments:

1. The assumption for comparing the temporally collected samples is that malaria case numbers have been reduced, which might lead to genetic isolation and structuring of parasite populations. It would be great if malaria epidemiology at the beginning and end of sample collection in the sites where samples were collected is clearly stated. It is possible that despite the overall reduction in malaria cases, some of the sites may represent hotspots where malaria epidemiology remained more or less unchanged over the time. As a result, this would make the parasite populations and genetics relatively stable over the time.
2. The inclusion of numerous SNPs for this type of analysis is a nice practice. However, the authors may want to separate those that are clearly under selection (such as EBA175 and AMA1), since these mutations are subject to strong immune selection and will have different evolutionary trajectories as compared to more neutral SNPs. 
3. More detailed comparison of the two Kenyan sites might be interesting to see whether gene flow between these sites exists, given that these sites are relatively closely located, yet separated by potential gene flow barriers (such as the rift valley).

I have read this submission. I believe that I have an appropriate level of expertise to confirm that it is of an acceptable scientific standard.

# Response

**Published:** 2017-08-29

**Authors:** Irene Omedo

**Affiliations:** KEMRI- Wellcome Trust Research Programme, Kenya

We are grateful for this review and the helpful comments and suggestions that have been made. We have included a point-by-point response (in bold) to the issues raised.

The assumption for comparing the temporally collected samples is that malaria case numbers have been reduced, which might lead to genetic isolation and structuring of parasite populations. It would be great if malaria epidemiology at the beginning and end of sample collection in the sites where samples were collected is clearly stated. It is possible that despite the overall reduction in malaria cases, some of the sites may represent hotspots where malaria epidemiology remained more or less unchanged over the time. As a result, this would make the parasite populations and genetics relatively stable over the time.

**The following statement has been added to show the changing epidemiology of malaria during the study period:**

**“Over the study period, malaria transmission, as measured by malaria slide positivity rate, fell from 56% in 1998 to 7% in 2009 in Kilifi ^1^, and rose slightly in Fajara and Brikama in the Gambia ^2^.”**

**There was no data showing temporal variation in malaria transmission in Rachuonyo South because the samples were collected in a single cross-sectional survey.**

The inclusion of numerous SNPs for this type of analysis is a nice practice. However, the authors may want to separate those that are clearly under selection (such as EBA175 and AMA1), since these mutations are subject to strong immune selection and will have different evolutionary trajectories as compared to more neutral SNPs. 

**We have included the following additional text to show the number of SNPs typed in EBA175 and AMA1:**

**“Separate analyses were also carried out for subsets of SNPs typed in EBA 175 (39, 36 and 20 SNPs in The Gambia, Kilifi and Rachuonyo South, respectively) and AMA1 (9 SNPs in The Gambia and 8 SNPs in Kilifi). Only 3 SNPs were genotyped in Rachuonyo South, so this SNP subset was not analysed separately.”**

****

**And expounded on the section describing the outcome of these analyses:**

****

**“In all study sites, separate analyses of EBA175 and AMA1 did not reveal qualitatively different results from the pooled analyses (supplementary figures 1- 3) and only the results of the pooled analyses are presented here.”**

More detailed comparison of the two Kenyan sites might be interesting to see whether gene flow between these sites exists, given that these sites are relatively closely located, yet separated by potential gene flow barriers (such as the rift valley).

**The current study focused on parasite movement and mixing within small, geographically-defined areas, and hence concentrated on analysing parasite genetics within individual sites. Furthermore, we did not have sufficient number of SNPs typed in these two populations to carry out a meaningful comparison. That said, your comment is important, and has been noted as a recommendation for future work, in the statement below:**

**“Furthermore, as transmission continues to decline and malaria programmes gradually shift their focus from control to elimination, the analysis of parasite gene flow between different transmission foci, e.g. Kilifi and Rachuonyo South will become increasingly important in informing the mitigation measures needed to prevent importation of parasites as a result of human movement and migration. These analyses were not carried out in the current study since the numbers of common SNPs between the two Kenyan sites was low, and we only had parasites from one timepoint in Rachuonyo South, hence we were unable to conduct an informative analysis of gene flow between sites”**

# Peer review: Referee response for version 1

**DOI:** 10.21956/wellcomeopenres.11628.r21343

**Published:** 2017-04-18

**Authors:** Christopher Delgado-Ratto

**Affiliations:** 1 Global Health Institute, University of Antwerp, Antwerp, Belgium

This is a study that used SNP genotyping data finely analysed to describe the geographic structuring of Plasmodium falciparum parasites at micro-epidemiological level in three regions from Gambia and Kenya.

 The authors were not able to compare the parasite populations among the study sites due to the samples were originally obtained for studies with different study designs (differences in sampling time, study population and design). The genetic diversity and clustering may not only be affected by geographic location and time but also by different ways of sampling the data. Say so, I appreciated that the authors focused in the population dynamics within the study sites.

 Regarding the hypothesis that exists gene flow within the study sites, gene flow models could be also useful to prove such genetic exchange of parasites. There are various software that may help on this matter, i.e. Migrate-n.

 Specific remarks:

 Conclusions section:

- This paragraph is not fully justified on basis of the results: “following mass-treatment campaigns we would predict that if residual foci of transmission are retained this will rapidly lead to reinfection of the wider community, and that parasites acquiring mutations conferring drug resistance or immunological escape will be rapidly spread at a micro-epidemiological level.”

I have read this submission. I believe that I have an appropriate level of expertise to confirm that it is of an acceptable scientific standard.

# Response

**Published:** 2017-08-29

**Authors:** Irene Omedo

**Affiliations:** KEMRI- Wellcome Trust Research Programme, Kenya

We would like to thank the reviewer for his comments, which helped to improve the manuscript. we have included our responses below (in bold):

Regarding the hypothesis that exists gene flow within the study sites, gene flow models could be also useful to prove such genetic exchange of parasites. There are various software that may help on this matter, i.e. Migrate-n.

**Migrate-n is a useful software for measuring migration rates between populations and is definitely useful for measuring gene flow within and between the different sites. It however, seems to need the parasites to be divided into populations and then to test for migration between these populations. Our current analyses aimed to step away from this requirement of specifying the populations a prior, and instead try to identify population structure based on clusters of genetically related parasites. The software also additionally requires the use of allele frequencies, while the current analyses focused on individual level genetic data. However, since such analyses may still be useful for identifying migration rates once distinct parasite populations have been identified, the following statement has been added in the discussion section.**

****

**“Finally, we used genetic data to show that there is high parasite movement and mixing within individual study sites. Additional analyses using gene flow models, e.g. as implemented in Migrate-N software, can be used to further validate our hypothesis of rapid gene flow and to confirm whether the parasites are part of a panmictic population or whether there exists underlying population structure, as well as to determine directionality of parasite movement between different populations, assuming that such populations can be identified within the region.”**

This paragraph is not fully justified on basis of the results: “following mass-treatment campaigns we would predict that if residual foci of transmission are retained this will rapidly lead to reinfection of the wider community, and that parasites acquiring mutations conferring drug resistance or immunological escape will be rapidly spread at a micro-epidemiological level.”

**The statement has been re-written as follows to clarify the message:**

****

**“On the other hand, based on the high level of parasite mixing observed at each study site, we would predict that ineffective application of control interventions such as mass drug administration that result in residual foci of transmission would lead to rapid re-infection of the wider community, and also that parasites acquiring mutations conferring drug resistance or immunological escape would spread rapidly at the micro-epidemiological level. This underscores the need for effective and sustained control until malaria elimination is achieved.”**

# Peer review: Referee response for version 1

**DOI:** 10.21956/wellcomeopenres.11628.r21345

**Published:** 2017-04-11

**Authors:** Cristian Koepfli

**Affiliations:** 1 University of California, Irvine, Irvine, CA, USA

This is a relevant study, assessing the ability to identify small-scale foci of transmission and parasite gene flow to surrounding areas based in SNP-typing. While it is overall clearly presented and well written, more detail, in particular in the results section, would help to better understand the data, and to assess its power.

 Specific comments:

 Abstract:

1. Please state how many samples and SNP markers were included in the final analysis.
2. I wonder whether “relatedness in space and time” is the correct term, or “distance in space and time” would be more appropriate.

 

 Results:

1. In the part on “Identification of geographical barriers to parasite movement” it would be useful to include the range of prevalence or MPF per pixel analyzed.
  The second paragraph of this part is difficult to follow, as the term ‘cluster’ is used consistently, without further indication on what the clusters represent. It would help to include a sentence describing that spatial clusters were analyzed based on the PCA values of all isolates found within the cluster. Thus, clusters of isolates differing from all other isolates were identified. The same is the case in the discussion. What sizes were the clusters identified, and how many haplotypes were included per cluster?
2. Figure 5: Given that for almost every pair of samples the number of days differs, how were the days for the different curves calculated? I assume each color represents a range, yet only a point estimate is given.
  Also, please indicate in brackets for each curve the number of samples included. For example, how many samples were available for the 1-day and 31-days analysis in The Gambia? Could the apparent reduction in SNP difference at 10 km be a chance finding due to limited sample size?
  Including the number of SNPs analyzed in each population would further help to interpret the data. E.g. it is interesting that in Rachuonyo South the proportion of different SNPs is approx. twice as high as in the other sites, yet this is only evident when Figure 5 is compared to Table 1.
  Would it be possible to include confidence intervals for the 1-day curves in the figure? This would help to understand the power of the data. For example, the statement in the abstract “Genetic relatedness of sample pairs is predicted by relatedness in space and time” suggests that genetic relatedness can be inferred, once the distance by space and time is known. This is however difficult to assess without more detail on the variance of the data.
3. In Table 2, what is the unit of the results showed? I assume it is SNP-difference/day (or SNP-difference/km), with days and distance log-transformed. Please state if/how data was transformed.

 

 Discussion:

1. Paragraph 3 of the discussion could be expanded. At what spatial scales was population structure found in previous studies (as compared to the approx. 50 km range of the present study)? Have any of these studies included relatedness? This information would help to assess the feasibility to identify foci of higher transmission, and to estimate the level of gene flow to surrounding areas in different transmission settings.
2. The number of SNP differences plateaus at approx. 1 km in The Gambia, 3 km in Kilifi, and increases up to 10 km in Rachuonyo South. Are there possible explanations for these differences due to the characteristics of the local parasite populations?

I have read this submission. I believe that I have an appropriate level of expertise to confirm that it is of an acceptable scientific standard.

# Response

**Published:** 2017-08-29

**Authors:** Irene Omedo

**Affiliations:** KEMRI- Wellcome Trust Research Programme, Kenya

We would like to sincerely thank the reviewer for taking time to review this work. We appreciate the comments and issues raised, and have addressed them on a point-by-point basis as indicated (in bold).

Abstract:

Please state how many samples and SNP markers were included in the final analysis.

**The following statement has been inserted in the abstract to show the number of samples and SNPs used in the final analysis.**

****

**"Using 107, 177 and 82 SNPs that were successfully genotyped in 133, 1602, and 1034 parasite isolates from The Gambia, Kilifi and Rachuonyo South district, respectively, we show that there are no discrete geographically restricted parasite sub-populations.”**

I wonder whether “relatedness in space and time” is the correct term, or “distance in space and time” would be more appropriate.

**“Relatedness in space and time” has been replaced with “distance in space and time”.**

Results:

In the part on “Identification of geographical barriers to parasite movement” it would be useful to include the range of prevalence or MPF per pixel analyzed.

The second paragraph of this part is difficult to follow, as the term ‘cluster’ is used consistently, without further indication on what the clusters represent. It would help to include a sentence describing that spatial clusters were analyzed based on the PCA values of all isolates found within the cluster. Thus, clusters of isolates differing from all other isolates were identified. The same is the case in the discussion. What sizes were the clusters identified, and how many haplotypes were included per cluster?

**The following statement has been added to show the MPF and parasite prevalence range analysed per pixel. The interquartile range of both measures at each pixel size has also been added.**

****

**“The range of MPF and parasite prevalence per pixel varied depending on the size of the pixels analysed. In the Kilifi population, MPF ranged from 0 – 100% (interquartile range (IQR) = 20%) for the 0.5km pixels; 0 – 100% (IQR = 14%) for the 1.0km pixels; 20 – 83% (IQR = 7%) for the 2km pixels; and 33 – 63% (IQR = 4.7%) for the 4km pixels. In the Rachuonyo South population, PCR positive prevalence varied from 0 – 75% (IQR = 19.4%) for the 0.5km pixels; 0 – 47% (IQR = 17.4%) for the 1.0km pixels; 3.5 – 35.8% (IQR = 14.3%) for the 2km pixels; and 6.2 – 33.4% (IQR = 7.8%) for the 4km pixels.”**

**The second paragraph under ‘spatial autocorrelation’ heading in the methods section has been expanded to indicate what clusters represent and how they are identified, and now reads as follows:**

**“Spatial scan statistics were calculated using SaTScan software and were run separately for each study site. The statistics involved running a purely spatial, retrospective analysis based on a normal probability distribution model using continuous variables (PC scores) and looking for areas with clusters of high PC scores. Latitude and longitude coordinates were used to represent the geographical locations of specific parasites, whereas principal component scores were used to represent individual parasite genotypes. During the analysis, a scanning window that gradually varies in size from including only a single homestead up to 50% of the population moves over the geographical space and at each window size and location, the ratio of parasites with high PCs inside the window versus outside the window is calculated. The window with the highest ratio is noted down as a cluster and its statistical significance is determined after accounting for multiple comparisons using random permutations.”**

**The number of samples contained in each significant cluster has been added in the results section and now reads as follows:**

**“In Kilifi, one cluster with a radius of 1.54 km (p=0.01) containing 15 parasites was detected, while in Rachuonyo South, a smaller cluster of genetically distinct parasites was detected with a radius of 0.5 km (p=0.001) containing 14 parasites”.**

**And the following section has been added in the discussion section to make it clear that the clusters were based on PC scores:**

**“However, using scan statistics we identified only two specific clusters of distinct parasite sub-populations based on PC scores, one in Kilifi and another in Rachuonyo South.”**

Figure 5: Given that for almost every pair of samples the number of days differs, how were the days for the different curves calculated? I assume each color represents a range, yet only a point estimate is given.

**The following statement has been added in the results section to show how the graphs were generated:**

****

**“Since the number of days differed for almost all parasite pairs, dummy data were included in the regression analysis to enable the generation of time-distance interaction graphs. For each study site, a distance range of 1 – 10km (with an interval of 0.1km between adjacent distances) was used. Temporal distance with 14 and 10 day intervals were assigned to parasite pairs in Kilifi and the Gambia, respectively, whereas time was not considered for the Rachuonyo South population. Constant SNP differences of 14, 10 and 8 were used for parasite pairs in Kilifi, Rachuonyo South and the Gambia, respectively.”**

Also, please indicate in brackets for each curve the number of samples included. For example, how many samples were available for the 1-day and 31-days analysis in The Gambia? Could the apparent reduction in SNP difference at 10 km be a chance finding due to limited sample size?

**The number of samples used to draw each curve has been noted in the Figure legend. We agree with the reviewer that the decrease past 10km is likely due to limited sample size and is there probably not significant.**

Including the number of SNPs analyzed in each population would further help to interpret the data. E.g. it is interesting that in Rachuonyo South the proportion of different SNPs is approx. twice as high as in the other sites, yet this is only evident when Figure 5 is compared to Table 1.

**The number of SNPs analysed in each population has been added to the figure legend.**

Would it be possible to include confidence intervals for the 1-day curves in the figure? This would help to understand the power of the data. For example, the statement in the abstract “Genetic relatedness of sample pairs is predicted by relatedness in space and time” suggests that genetic relatedness can be inferred, once the distance by space and time is known. This is however difficult to assess without more detail on the variance of the data.

**Figure 5 has been regenerated to show the confidence intervals for the 1-day curves.**

In Table 2, what is the unit of the results showed? I assume it is SNP-difference/day (or SNP-difference/km), with days and distance log-transformed. Please state if/how data was transformed.

**The following statement has been added to Table 2 to show the units of measurement of the results.**

**“Values represent the change in the number of SNP differences between parasite pairs per day (time), per kilometre (distance) and per day/kilometre (time-distance interaction). Time, distance and the product of time and distance (time-distance interaction) were log transformed prior to running the regression analyses.”**

Discussion:

Paragraph 3 of the discussion could be expanded. At what spatial scales was population structure found in previous studies (as compared to the approx. 50 km range of the present study)? Have any of these studies included relatedness? This information would help to assess the feasibility to identify foci of higher transmission, and to estimate the level of gene flow to surrounding areas in different transmission settings.

**The paragraph has been expanded and now reads as follows:**

**“Lack of genetic structuring of parasite populations observed in this study is indicative of a population that is well mixed. This observation of a highly mixing parasite population is in agreement with results of similar studies of parasite genetic diversity and population differentiation using microsatellites ^52^^, ^^54^^, ^^55^ , immune selected genes ^56^^, ^^57^ and SNPs ^58^ . These studies were carried out in parasite populations from different geographical regions representing a diverse range of transmission intensities from highest in Africa and oceania, intermediate in Southeast Asia, and lowest in south America. However, other studies have shown population structure when looking at the same population ^48^^, ^^59^^– ^^61^ , although these analyses were carried out on larger geographical scales than those analysed here and mostly involved analyses at provincial, country or continental levels. Population structure was most evident in regions with low transmissiion intensities such as south America or southeast Asia, and less evident in Africa where transmission intensity is much higher.”**

The number of SNP differences plateaus at approx. 1 km in The Gambia, 3 km in Kilifi, and increases up to 10 km in Rachuonyo South. Are there possible explanations for these differences due to the characteristics of the local parasite populations?

**Although the exact reason for the plateau observed is currently unknown, the following statement has been added to postulate possible reasons for the observation:**

**“The number of SNP differences were seen to plateau at approximately 1km in the Gambia, 3km in Kilifi and 10km in Rachuonyo South. This may be attributed to the characteristics of the local parasite population, which in turn may be explained by the distribution of human settlement in the areas sampled, for example in the Gambia, homesteads tend to be clustered together in distinct, autonomous villages whereas in Rachuonyo South there is a denser and more uniform pattern of human settlement over the study area, enabling the interaction of parasites over a much larger distance.”**

# Peer review: Referee response for version 1

**DOI:** 10.21956/wellcomeopenres.11628.r21178

**Published:** 2017-03-29

**Authors:** Michel Tibayrenc

**Affiliations:** 1 Maladies Infectieuses et Vecteurs Ecologie, Génétique, Evolution et Contrôle (MIVEGEC), Institut de Recherche pour le Développement (IRD), Montpellier, France

This is a fine population genetic analysis of 3 samples taken in Gambia and Kenya, relying on the typing of 5199 samples by 276 SNPs. I have little to say about this work, which uses sound approaches and yields clear conclusions. A few remarks:

1. How can heterozygous genotypes be detected in haploid populations of the parasite?
2. As noted by the authors themselves, using 276 SNPs is rather limited. Genetic studies dealing with human populations at nowadays routinely rely on 500000 SNPs or more. One main feature of such studies is that microgeographical structures are deteted mostly from low frequency variants and rare variants, which of course are undetectable when using a limited set of markers. Moreover, these low frequency and rare variants are supposed to be highly relevant for phenotypic expression, in particular disease susceptibility and are largely responsible for recent and localized evolution in human populations. (see for example Leslie et al. (2015) ^1^). It is most probable that these patterns exist in parasite populations too. The authors should discuss this point more, since it is probably one of the main avenues of future researches in microbiology.

I have read this submission. I believe that I have an appropriate level of expertise to confirm that it is of an acceptable scientific standard.

## References

[1] LeslieSWinneyBHellenthalGDavisonDBoumertitADayTHutnikKRoyrvikECCunliffeBWellcome Trust Case Control Consortium 2International Multiple Sclerosis Genetics ConsortiumLawsonDJFalushDFreemanCPirinenMMyersSRobinsonMDonnellyPBodmerW: The fine-scale genetic structure of the British population. *Nature*.2015;519(7543) : 10.1038/nature14230 309-14 10.1038/nature14230 25788095PMC4632200

# Response

**Published:** 2017-08-29

**Authors:** Irene Omedo

**Affiliations:** KEMRI- Wellcome Trust Research Programme, Kenya

We would like to sincerely thank the reviewer for taking time to review our work. We appreciate your comments, and have included a point-by-point response to them as follows (our responses are in bold).

How can heterozygous genotypes be detected in haploid populations of the parasite?

**The use of the words “homozygous” and “heterozygous” in the context of haploid organisms has been clarified to mean single parasite genotype infections and mixed parasite genotype infections, respectively.**

As noted by the authors themselves, using 276 SNPs is rather limited. Genetic studies dealing with human populations at nowadays routinely rely on 500000 SNPs or more. One main feature of such studies is that microgeographical structures are deteted mostly from low frequency variants and rare variants, which of course are undetectable when using a limited set of markers. Moreover, these low frequency and rare variants are supposed to be highly relevant for phenotypic expression, in particular disease susceptibility and are largely responsible for recent and localized evolution in human populations. (see for example Leslie et al. (2015) ^1^). It is most probable that these patterns exist in parasite populations too. The authors should discuss this point more, since it is probably one of the main avenues of future researches in microbiology.

**The following additional information has been added in the discussion section to address this point:**

**“Advances in sequencing technologies have increased the use of whole genome sequence data in the analysis of *P. falciparum* parasite population genetics, and this has led to the identification of hundreds of thousands of SNPs, most of which are present at very low frequencies especially in African parasite populations ^3^. Additional analyses will require the use of whole genome sequence data to identify rare variants and distinguish between closely related parasites, thus allowing parasite population structure to be analysed at fine spatial scales.”**
